# Epitaxial diamond-hexagonal silicon nano-ribbon growth on (001) silicon

**DOI:** 10.1038/srep12692

**Published:** 2015-08-04

**Authors:** Y. Qiu, H. Bender, O. Richard, M.-S. Kim, E. Van Besien, I. Vos, M. de Potter de ten Broeck, D. Mocuta, W. Vandervorst

**Affiliations:** 1 Imec, Kapeldreef 75, Leuven, Belgium; 2 Instituut Kern-en Stralings Fysika, K.U.Leuven, Leuven, Belgium

## Abstract

Silicon crystallizes in the diamond-cubic phase and shows only a weak emission at 1.1 eV. Diamond-hexagonal silicon however has an indirect bandgap at 1.5 eV and has therefore potential for application in opto-electronic devices. Here we discuss a method based on advanced silicon device processing to form diamond-hexagonal silicon nano-ribbons. With an appropriate temperature anneal applied to densify the oxide fillings between silicon fins, the lateral outward stress exerted on fins sandwiched between wide and narrow oxide windows can result in a phase transition from diamond-cubic to diamond-hexagonal Si at the base of these fins. The diamond-hexagonal slabs are generally 5–8 nm thick and can extend over the full width and length of the fins, i.e. have a nano-ribbon shape along the fins. Although hexagonal silicon is a metastable phase, once formed it is found being stable during subsequent high temperature treatments even during process steps up to 1050 ºC.

Silicon normally crystallizes in the diamond cubic (dc) structure, which corresponds to the zinc-blende structure with single atom type. Cubic Si has an emission around 1.1 eV corresponding to an indirect band gap with low intensity radiation. Therefore it is not a material of interest for the active regions of optoelectronic devices. It is mainly used for microelectronic devices and for solar cells. However, there are at least 13 phases of silicon reported, most of which only form under high pressure conditions and are otherwise unstable^1^. The existence of Si in different crystal structures with different electronic properties offers increased flexibility in the design of future Si-based devices^2^. Among them, hexagonal silicon with lonsdaleite structure, which corresponds to the wurtzite structure with single atom type, and which is also referred to as diamond-hexagonal (dh) Si, Si IV (A) or 2H Si, attracted lot of attention during last decade. In 2002, Raffy *et al.* predicted that for Si the fundamental energy gap decreases with increasing hexagonality of the polytype^3^. It is recently shown experimentally that dh-silicon can emit visible light (direct transition at ~1.5 eV) with about two orders of magnitude higher efficiency than dc Si^4^,^5^ as well as near-infrared light^5^ (indirect band gap at ~0.8 eV). dh-Si, with P6_3_/mmc space group, 4 atoms per primitive unit cell and approximate lattice parameter of  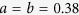
nm and 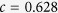
nm, was reported firstly by Wentorf and Kasper^6^ in 1963. It was formed by heating Si samples recovered from previous high-pressure experiments up to 200 ^o^C–600 ^o^C for times between 30 minutes and 3 days under atmospheric pressure^6^. This phase has the same density as the dc-Si phase and is known as metastable with respect to the diamond phase^7^ and possibly stable up to ~530 ^o^C^8^. dh-Si is also found in the process of plastic deformation under indentations in the temperature range 400 ^o^C–700 ^o^C^9^,^10^,^11^,^12^,^13^. In addition, dh-Si was observed in ion-implanted silicon^12^ and also after long time annealing at 450–750 °C of oxygen rich Si^14^,^15^,^16^. Zhang *et al.* have shown that thin films of polycrystalline dh-Si can be deposited using ultraviolet laser ablation at low pressure from a cubic silicon target^17^. Since 2005, the presence of dh-Si phase is reported and intensively studied in silicon nanowires (NWs) synthesized by the vapor-liquid-solid growth method with gold^4^,^5^,^18^,^19^,^20^,^21^,^22^,^23^ or copper^24^ nano-catalyst particles. Single crystal long-term stable dh phase is realized in boron-doped Si NWs^4^. Both 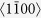
4,5 and 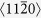
18 directions are reported along the axis of the wires with 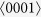 oriented across the NWs. Recently nanocrystals with a dh-Si structure are grown in carbide-based film using a plasma-enhanced chemical vapor deposition method^25^.

The first transmission electron microscope (TEM) investigation of dh-Si was performed by Eremenko and Nikitenko^9^. Based on an electron diffraction study, they were able to determine the habit plane between the dc and dh phases to be 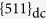 and the relationship between dc and dh Si as 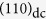//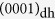 and 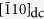//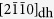. It is proposed that the 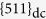 habit plane is the continuation of the coherent twin boundary between primary and secondary twins and that the phase transformation from dc to dh happened in the region where an inverse twinning shear has taken place, i.e. a twin-twin intersection or dislocation mechanism^13^,^26^,^27^,^28^. In addition, Cerva found another relationship between dc and dh Si due to polytypic transformation in polycrystalline Si layers: 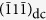//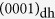, 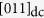//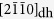
29. This relationship is also reported in some nanowires studies^18^,^20^,^21^.

In this work a new method to form dh-Si is discussed. The phase is characterized in detail with high resolution scanning transmission electron microscopy (HR-STEM) and chemical analysis. The growth procedure is based on FinFET device processing for the sub-14 nanometer technology node. Under specific conditions, dh-Si nano-ribbons can form at the base of the fins during the oxide fill step. The phase transition generally results in an outward shift of the fin which however maintains its crystal alignment relative to the substrate. Once formed, the dh-Si ribbon is stable during further processing including implantation anneal, epitaxial growth and metallization step. The phase and interface characterization and formation mechanism leading to this phase transition are discussed in detail. As the phase is in a specific epitaxial relation to the substrate and forms at well-defined positions in the devices, it opens new possibilities for integration of optoelectronic applications in standard Si-based device technologies.

## Results

**Figure 1:**
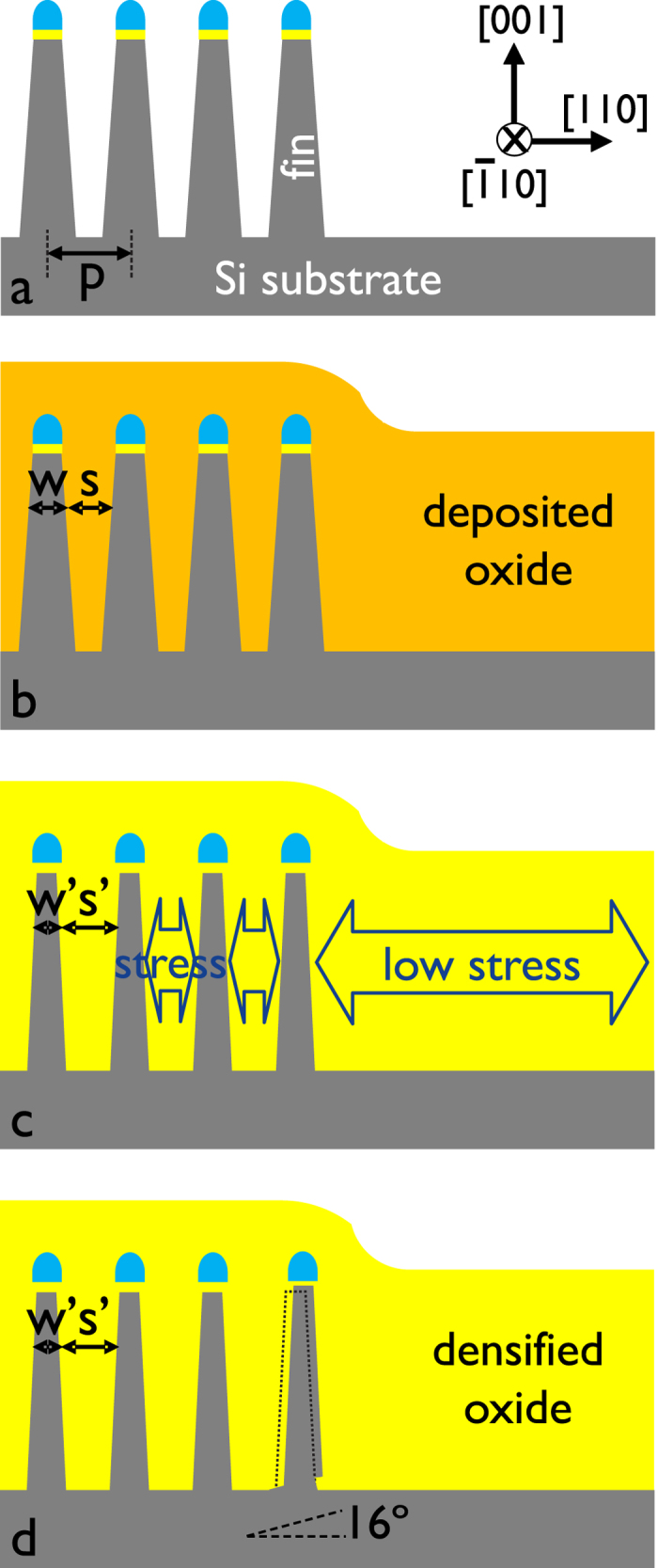
Schematic of the major processing steps involved for the dh-Si formation. Fins with 45 nm pitch p are etched in the silicon (**a**), the spaces are filled with chemical vapor deposited (CVD) oxide (**b**) which is subsequently densified (**c**) during which step the geometry change at the bottom of the outer fin happens (**d**). The schematic illustrates the edge of a group of fins. The nitride cap (blue) acts as hardmask during the Si fin etch. More details on the oxidation step are illustrated on Supplementary Fig. S1 and S2 online.

**Figure 2:**
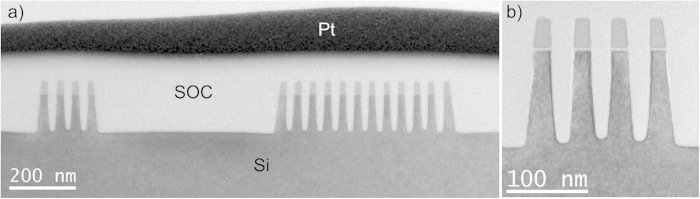
Bright field (BF) TEM images of groups of fins after etching. (**a**) An overview of the groups of both 4 fins and 12 fins, (**b**) the group of 4 fins with higher magnification. The spacings are filled with Spin-On-Carbon (SOC) for TEM specimen preparation. The nitride/oxide hardmask on the fins is used for the fin etching.

**Figure 3:**
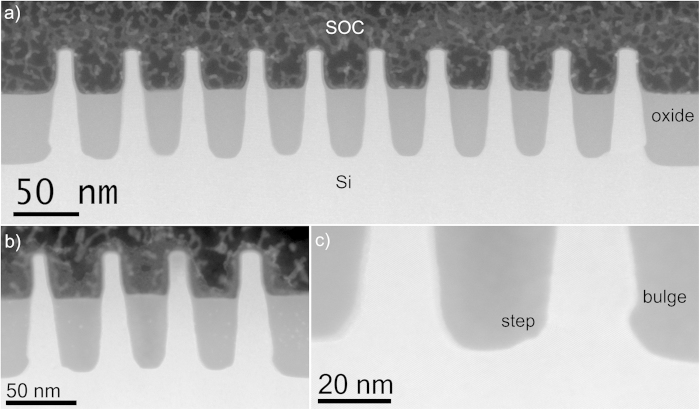
HAADF STEM images after oxide fill and anneal. Both in the group of 10 fins (**a**) and of 4 fins (**b**) the outer fins are shifted outwards i.e. the spacing increased and steps and bulges are formed as illustrated in the higher magnification image (**c**). These images are taken after further process steps where the nitride cap is removed and the oxide is recessed between the top of the fins. These steps have no effect on the geometry change of the fin induced by the oxide densification step.

**Figure 4:**
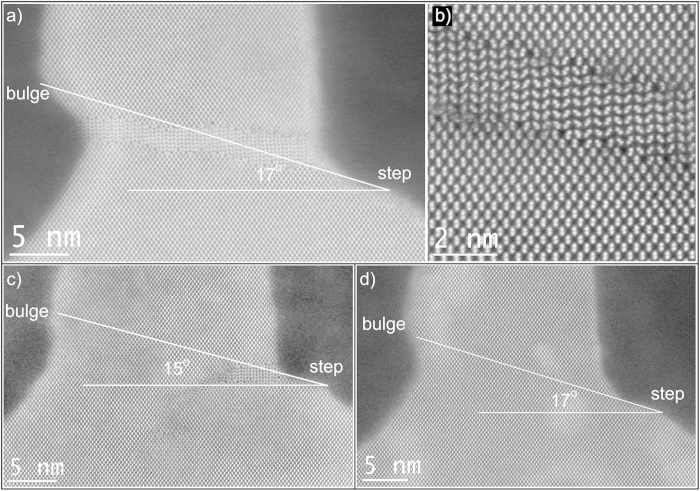
High resolution HAADF STEM images of the 3 main configurations at the bottom of the outer fins. (**a**) A defect/slab across the full fin width, with a further zoomed in HR-HAADF STEM image of the slab area in the top right image (**b**). (**c**) Defect at the base of the inner side of the fin and (**d**) no defect but similar as in (**a**) and (**c**) a step on the bottom of the spacing and a bulge at the outside of the fin.

The important fin processing steps are schematically illustrated in Fig. 1. The TEM investigations are performed after Si fin etching (Fig. 1a), after the oxide fill and densification (Fig. 1d) and after further processing steps of the device up to first level metallization. A TEM image of groups of 12 and 4 fins after the Si etch is presented in Fig. 2a, while Fig. 2b zooms in at the group of 4 fins with higher magnification. The spacings between the fins on these images are filled with Spin On Carbon (SOC) for TEM specimen preparation. The fins are well defined with a smooth profile at the base of their sidewalls. All fins show the nominal 45 nm pitch. Due to the etch the outer fins are slightly asymmetric. The morphology of the outer fins is modified after the oxide fill and densification as illustrated in Fig. 3. In both groups of fins, the outer spacings are enlarged and the outer fins moved outwards compared to the configuration after the etch. The width of the fins is slightly reduced by the oxidation. The profile at the bottom and the pitch of the inner fins are unchanged. The outer fins appear wider than the inner fins and have a typical bulge at the outer bottom and a step on the silicon on the inner side. The direction from step to bulge is nearly 16º inclined upwards (Fig. 3c and 4) to the outside of the group of fins, i.e. corresponds to a {115}_dc_ plane. Step and bulge have similar widths and indicate an outward shift of the fin along this plane (Fig. 1d). Zooming in at the bottom of the outer fins after oxidation and annealing, shows 4 possible configurations which occur in a ratio 45/15/30/10 in the over 300 studied finfet structures: a defect across the full fin width (Fig. 4a), a partial defect (Fig. 4c), no defect but bulge and step present with similar dimensions as for the defected cases (Fig. 4d), and rarely no defect and no clear bulge or step. The defects consist of a slab of material of different phase with a thickness of about 5 to 8 nm embedded between dc-Si. The full defects run from the step to a position below the bulge as illustrated in Fig. 4a. The width of the defected region is narrower than the Si below and above (including the step and bulge respectively). Figure 4b shows a high angle annular dark field (HAADF) HR-STEM image taken at 300 kV. The dumbbell structure which can be seen in the silicon substrate and fin, is also present inside the defect with similar brightness of the atomic columns, i.e. same composition but different structural arrangement of the columns. Energy dispersive X-ray spectroscopy confirms that the material consists of Si only. The partial defects are always situated next to the step at the inner side of the outer fins as illustrated in Fig. 4c.

**Figure 5:**
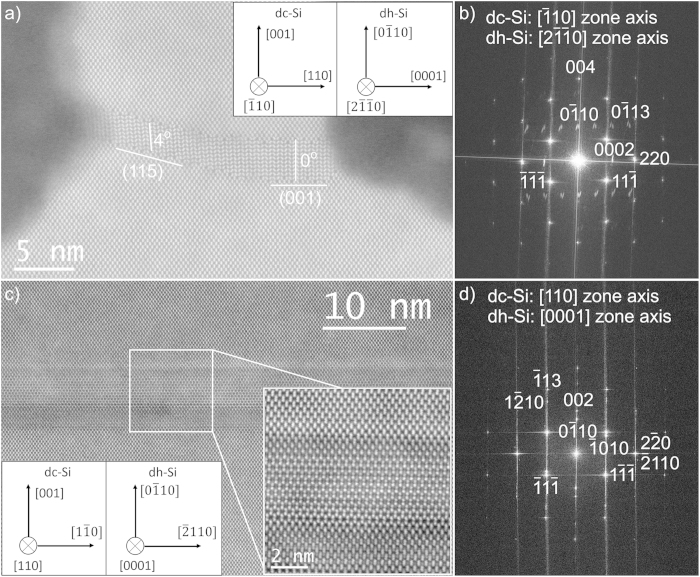
HR HAADF STEM images of the slab material observed across and along the fin. The crystallographic relationship between the dc-Si and dh-Si lattices as can be derived from the HR-STEM image (**a**) and the fast Fourier transform (FFT) of the image (**b**) is illustrated by the schematic in (**a**) and the indexing of the spots (**b**). The bottom image (**c**) shows a cut parallel with an outer fin with insert at bottom left of the lattice relationship and insert at bottom right presenting the dh-Si slab region at higher magnification, and the corresponding FFT with indexing of the spots (**d**).

Detailed crystal structure analysis of the slab material is shown in Fig. 5. The atomically resolved HAADF STEM image viewed along the fin direction (Fig. 5a) shows that the defect consists of a single crystalline material which is in epitaxial relationship to the dc-Si of substrate and fin. The crystal structure is analyzed from the Fourier transform (FFT) of the image (Fig. 5b). The sharp bright spots in the FFT pattern are due to the dc-Si of substrate and fin along 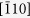 zone axis. The streaked spots with lower intensity are from the slab material. The spots are doubled and elongated due to a rotation of the lattice in the left and right part of the defect. The reflections can be indexed as shown in Fig. 5b as diamond-hexagonal silicon observed along its 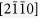 zone axis. The relationship between dc-Si and dh-Si can be summarized as 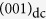//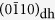, 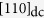//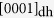 and 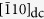//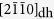, which is the same epitaxial relation as observed for diamond-hexagonal silicon in indentation structures^9^. The lattice parameters a and c are determined from the FFT pattern as 0.38 nm and 0.63 nm respectively with the ratio of c/a = 1.65, i.e. very close to the ideal value for dh-Si as can be calculated based on the dc-Si parameters. The majority of the analyzed defects shows a stepped configuration as on Fig. 4a and 5a, i.e. part of the defect at the inner side of the fins show 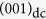 interfaces and next part has sloped interfaces parallel 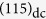. Some defects show additional steps between both kinds of interfaces, while only a few ones are observed that show almost exclusively the 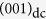 interface. The lattice in the horizontal defect parts is exactly aligned with the above mentioned relationship, i.e. has 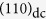//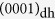, while a rotation of ~4° around 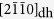 is present in the defect regions with 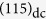 interface plane. This rotation can be related to the different interface structure between the dc and dh lattices at both interfaces (Supplementary Fig. S3–S5 online). Such rotation is also reported by Tan *et al.*^12^ for their model of dh-Si with 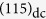 interfaces formed in As ion implanted silicon and for 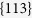 defects in ion implanted Si^30^.

An atomically resolved HAADF STEM image at the slab area of a sample cut parallel with an outer Si fin is presented in Fig. 5c. The dh-Si ribbon is magnified further as insert at the bottom right. In the dc-Si of fin and substrate the typical dumbbell pattern is observed, while in the defect region clearly the regular hexagonal pattern corresponding to the dh-Si atomic columns along 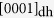 can be seen. The visibility of the latter pattern will only be ideal for defect parts that are observed exactly along the 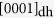 zone axis, and with the interface 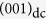//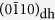 extending on-edge through the full TEM specimen thickness. In view of the defect dimension and the step configuration, even in a very thin sample, this condition will only rarely be fulfilled. Superposition of dc and dh lattices in the viewing direction will result in vertical streaking of the atomic columns. In the inclined defect parts with 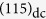 interfaces, this streaking will be further enhanced by the 4º rotation of the dh lattice. Therefore the clear visibility of the dh-lattice is limited to smaller areas along the defect. Observations at different positions along the length of the fin show that the dh-lattice can be recognized in regions with several hundred nanometer length, i.e. nano-ribbons of dh-Si are present at the base of the fins. The dh-regions are separated by parts of the fin that appear as dc-Si, which will correspond to the 3^rd^ (or 4^th^) kind of configuration as observed in the across fin views, i.e. step/bulge without defect. The relationship between dc-Si and dh-Si for the observation of the cut parallel with fin, is presented by the insert at the bottom left of Fig. 5c and the FFT shown in Fig. 5d. It fully agrees with the epitaxy derived from the cut perpendicular to the fin. The combined information from both observation directions is fully conclusive for the dh-Si nature of the defect and excludes the possibility of presence of inclined twin defects as discussed for dh-Si nanowires^4^,^5^.

**Figure 6:**
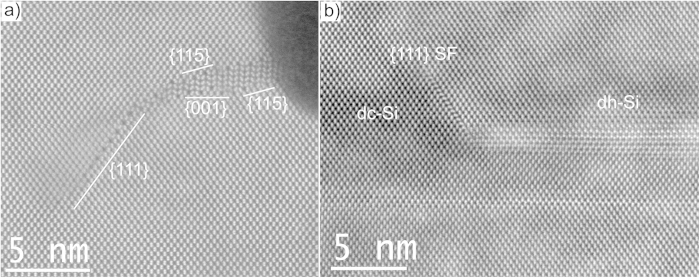
HR HAADF STEM images at dh-Si edges. Sample cut perpendicular to a fin with partial defect (**a**) and sample cut parallel with an outer fin (**b**). Both show the presence of defects in 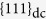 planes at the end of the dh regions.

Many partial dh-Si regions show a planar defect at their ends in a {111}_dc_ plane directed towards the silicon substrate (Fig. 6a). The lattice resolution is not always clear at these defects, but where interpretable they correspond to stacking faults. In TEM observation mode these defects are often strongly electron beam sensitive, i.e. grow during imaging. Stacking faults bonded by a 1/6[112] Shockley partial are also present at the dc/dh transition observed in samples cut parallel with the fin (Fig. 6b).

## Discussion

The crucial process steps that lead to the diamond-hexagonal silicon formation are schematically illustrated in Fig. 1. After the Si etch of the fin structures, the sidewalls have a smoothly varying profile and the silicon is defect free. The fin morphology is unchanged during the oxide fill step but is modified during the subsequent wet oxygen anneal. The oxidation process is discussed in more detail on Supplementary Fig. S1 and S2 online. Major change due to the oxidation is an outward shift of the outer fins characterized by an increase of the width of the last spacing, the formation of a bulge on the outside and of a step on the silicon at the bottom of the outer spacing. In about 60% of the outer fins this goes together with the formation of a hexagonal silicon slab over the full or partial width of the fin. Most samples investigated are further cured in N_2_ at higher temperature and received also additional temperature treatments during the further processing of the devices. All these subsequent steps are found to have no impact on the presence or morphology of the hexagonal silicon ribbons. Hence the key process step that causes the phase transition to hexagonal silicon is the wet oxidation while the formed dh-phase is stable during further thermal steps. The wet oxidation simultaneously densifies the deposited oxide and oxidizes the sidewalls of the fins, i.e. the fin width *w* decreases and the spacing *s* increases with the same amount. The conversion of Si to SiO_2_ results in a volume expansion by a factor 2.2, i.e. *t*_*ox*_ *= 2.2t*_*Si*_ with *t*_*ox*_the oxide thickness and *t*_*Si*_ the thickness of the consumed Si. The strain *ε*_*ox*_ in the oxide can be estimated from this volume expansion as
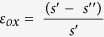
with *s’* the physical width of the spacing after the oxidation, i.e. *s’ = s + 2 t*_*Si*_ and *s” = s + 2 t*_*ox*_ the oxide width that has to fit in the physical width *s’*. Based on Hooke’s law for uniaxial load it allows to calculate the stress as *σ*_*ox *_*= E ε*_*ox*_with *E* the Young’s modulus of the oxide. At the given oxidation temperature, the oxide is not viscous and is therefore not pushed upwards out of the trench. Hence the mechanical stress is horizontally exerted on the sidewalls of the fins and is maximum near the bottom (Fig. 1c, Supplementary Fig. S1e online). The fins are slightly tapered after the etch so that the initial spacing *s* varies from about 25 nm at the top to 10 nm at the bottom of the fin which leads for the applied oxidation conditions to stress of about 19 GPa and 35 GPa respectively. Part of the volume expansion will however be compensated by the densification of the deposited oxide. If half of the expansion is accommodated by the densification, the stress will be reduced by nearly a factor two, and is therefore still considerable. For inner fins the inward and outward stress on both sides is quasi the same. However, for the outer fins the outward stress on the inner side of the fin is much larger than the inward stress on the outer side because the space to the next structure is in micrometer range and the estimated stress at that side remains below 1 GPa (Fig. 1c). Hence, the phase transformation and outwards shift of the outer fins can be related to the strain/stress from the unbalanced expansion during the wet oxidation step of the wide and narrow oxide windows next to the outer fins. The estimated stress levels are in the range needed to have a transition from dc-Si to dh-Si and it can be expected that the transformation will be initiated near the bottom of the fin where the stress is the largest. Once the transformation is finished and the fin shifted, the stress in the outer fin is released and no further transformation will occur during further processing steps that are not leading to additional oxide expansion. Moreover the high temperature N_2_ anneal after the oxidation step will allow vertical viscous flow of the oxide so that a general stress reduction occurs.

The bulges and inner steps have similar sizes for all 3 major types of configurations at the bottom of the outer fins. The occurrence of similar fin shifts also without presence of dh-Si and with partial dh-Si slabs indicates that the partial dh-regions are not the nucleation state of the full dh-slabs. A transformation of mass from the inner side to the outer side of the fin could occur by dislocation nucleation at inner side, glide through the fin width and annihilation of the dislocation at the outside. However the bulge should then be situated much higher so that the step to bulge plane would correspond to a (111)_dc_ glide plane. Dislocation formation and glide in Si at the low densification temperatures is however very unlikely and below 700 ºC, plastic deformation in Si occurs by other mechanisms such as twinning and as shown in the present study by phase transformation. Moreover, dislocation creation and glide cannot lead to dh-Si formation.

Transformation of dc- to dh-Si in indentations experiments^9^,^13^,^26^,^28^ is explained as a stress induced martensitic transformation involving shear at the intersection of the secondary twins which are generally present in high density in such indents^13^,^26^. In our case, only single stacking faults are observed at the edges of the dh-regions but never extensive twinning is observed. Therefore such martensitic transformation mechanism seems less likely to explain the dh-Si formation. A model for direct transition of dc to dh-Si by application of high stress on the (110)_dc_ plane was proposed by Tan *et al.*^12^. It involves bond breaking and the formation of new 6 atom rings which after relaxation result in dh-Si with the 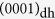 plane parallel 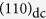. The transformation results in a 18% volume contraction in the 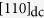 direction and equal expansion along 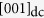 while no volume change occurs in the 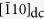 direction. Such mechanism could explain the presence and size of the steps on the inner side of the spacing and the reduced width of the dh-Si slab compared to the dc-Si above and below. To explain the shift of the fin also in cases of partial or no dh-Si, a reverse transformation mechanism should occur once the stress is released so that the dc lattice is recovered. Although dh-Si is generally considered to be a metastable phase^7^, the observed stability of the dh-Si slabs during further processing is in contradiction with such reverse transformation. An alternative process that overcomes this problem could be a dc- to dh-Si transformation that involves a cycle through several high pressure Si phases as observed in high pressure and indentation experiments. In such experiments an irreversible transition to β-tin Si (Si II) is observed for pressures above 12 GPa^10^,^11^ which is in the estimated pressure range at the bottom of the fins. The high pressure β-tin phase shows a 30% volume reduction per Si atom compared to dc-Si which can explain the formation of the step and reduction of the lateral width of the final dh-Si slab compared to the Si above and below. Upon unloading, the β-tin phase relaxes through a sequence of Si phases. In a combined Raman and TEM study with *in-situ* anneal of nanoindents in Si, Ge *et al.*^10^ showed that the phase sequence for slow reduction of the stress involves Si II (tetragonal ) → Si XII (rhombohedral) → Si III (bcc) → (Si XIII) and then coexistence of dh-Si (Si IV) and amorphous Si. At the oxidation temperature used for the densification step of the oxide fill a thin region of amorphous Si confined between the dc-crystalline substrate and fin can easily epitaxially regrow. Therefore following such phase sequence the final state could be the coexistence of dc- and dh-Si. The formation of either phase in the transition region might be favored by the local shape and interfaces of the intermediate phases. As these phases are metastable and quickly anneal out above 300 ºC^10^,^11^, they will not be present anymore after the oxide densification step and no direct evidence of this phase sequence is observed in our experiments. Nevertheless, such process could explain the presence of step/bulge with partial or no hexagonal silicon slab combined with stability of the hexagonal phase during further processing.

Most dh-Si slabs show a change of the interface with the dc-Si from inner to outer side of the fin. Whereas the 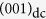//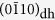 interface corresponds to the perfect alignment of 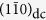 with 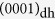, the 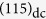 to 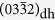 interface implies a rotation over 3.5º as experimentally observed. The latter interface is discussed in literature^12^,^13^ whereas the first case is not reported before. Both interface structures can be modelled with 5 and 7 atom membered rings without any dangling bonds (Supplementary Fig. S3 and S4 online) and show over a nearly 2 nm distance common lattice positions with only a small misfit (Table 1). However for the 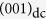//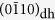 interface the reshuffling of the bonds needs to be spread over 2 atomic planes resulting in a stepped dc/dh interface which will be less stable over longer distances (Supplementary Fig. S3 online). Steps at the interface may then result in a switch to the more easily formed 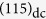 interface.

The formation of dh-Si requires sufficiently high stress to initiate the phase transformation and therefore does not occur for conditions that lead to lower stress, e.g. by applying less severe oxidation conditions (lower temperature, shorter oxidation time) or with larger initial spacing. As the dh-Si is situated at the base of the fins, it has no direct impact on the electronic properties of the devices that are processed on top of the fins as they only depend on the upper ~50 nm part of the fins. On the other hand by controlling the oxidation conditions and relative spacings on both sides of the fins, conditions that favor the formation of a dh-Si ribbon can be optimized. As the position and crystallographic orientation of the resulting dh-Si are well controlled, this opens possibilities to combine the optical properties of this phase in opto-electronic devices with advanced FinFET based nano-electronic devices. For example, by gradually increasing the spacing between the fins one could generate a series of parallel dh-Si nano-ribbons which could be used as waveguides in the devices. Further investigation of both design and processing conditions is needed to explore the possibilities of such applications. Conditions that lead to increased dh-Si volumes (thicker, higher density of ribbons) are also needed to proof the optical functionally of the material by cathodoluminescence, the sensitivity of which turned out to be insufficient for the present material.

## Methods

### Bulk FinFET process

The investigated FinFET structures are taken from a range of wafers processed up to different end-steps for process and device optimization. After the Si etch of the bulkfin structures the spacings between the fins are filled with a CVD oxide (O_3_/TEOS)). To reduce the void in between the fins and to densify the deposited oxide a wet oxidation anneal at low temperature (700–750 ºC) or a combination of wet oxidation anneal and high temperature inert cure (>1000 ºC) are applied. Figure 1 presents a schematic drawing of the process steps involved relevant to this investigation. The major steps are similar as discussed by Redolfi *et al.*^31^ but in the present work self-aligned dual patterning technology is used to obtain 14 nm node fin dimensions with fin pitch of 45 nm^32^. The investigated fin structures consist of groups of 4, 10 or 12 fins with constant pitch. Due to the etch, the sidewalls are sloped so that the spacing typically varies after etch from 25 nm at the top to 10 nm at the bottom. The groups of fins are separated by distances of more than 500 nm (Fig. 2a). The fins have lengths of 250 to 2500 nm and heights in the range of 100–130 nm.

### TEM specimen preparation

All the TEM samples are prepared by Focused Ion Beam (FIB, FEI Helios450HP) with the *in-situ* lift-out technique. To protect the sample surface during the ion milling a Spin-On-Carbon (SOC) layer is deposited on the full wafers or wafer pieces. This cap material fills high aspect ratio topography well and weakly planarizes the surface. The region of interest is further locally capped in the FIB with electron beam or ion beam deposited Pt. The major milling is done with a 30 kV Ga ion beam while the milling progress is controlled with the scanning electron microscope. Final milling to minimize the damage layer on the specimens is performed with 5 kV Ga ion beam. The typical TEM specimen thickness is ~50 nm. Most samples are prepared across groups of 4, 10 or 12 fins. In addition also TEM specimens are prepared parallel with the fins so that only the outer fin of the groups is included in the TEM specimen.

### TEM analysis

Investigation by transmission electron microscope is performed with FEI Tecnai F30 or Titan 60–300 Cube in TEM or STEM imaging mode. The high resolution STEM images are acquired in the double corrected Titan system with the high-angle annular dark field (HAADF) detector generally at 120 kV, unless specified otherwise. This condition allows high resolution that resolves the Si-dumbbells without noticeable beam damage in the specimens. The images are acquired with a screen current of 0.1–0.2 nA, a convergence angle of 22 mrad and an inner collection angle of 50 mrad. HAADF contrast, also known as Z-contrast, can provide directly not only the location of the column of atoms but also information on the local composition at atomic scale. However, resolution can be further improved by using 300 kV.

## Additional Information

**How to cite this article**: Qiu, Y. *et al.* Epitaxial diamond-hexagonal silicon nano-ribbon growth on (001) silicon. *Sci. Rep.* **5**, 12692; doi: 10.1038/srep12692 (2015).

## Supplementary Material

Supplementary Information

## Figures and Tables

**Table 1:** Distances between coincident lattice positions in the dc to dh interface and lattice mismatch.

**Interface dc//dh**	**Distance dc**	**nm**	**Distance dh**	**nm**	**Ratio dh/dc**	**Interface structure**
(001)//(0-110)	5/2 [110]	1.9192	3 [0001]	1.8951	0.9874	5 and 7 atomic rings in dual plane
(115)//(03-32)	1/2 [552]	1.9944	[01–13]	2.0083	1.0069	5 and 7 atomic rings in single plane
Both interfaces, distance along fin	1/2 [1–10]	0.3838	1/3 [2-1–10]	0.3837	0.9997	

Calculated with dc-Si : a = 0.54282 nm–dh-Si : a = 0.3837 nm, c = 0.6317 nm^8^. Corresponding interface models are discussed in the supplementary information online.
