# Supplementary material for: Epitaxial diamond-hexagonal silicon nano-ribbon growth on (001) silicon
Source: Sci Rep. 2015 Aug 4;5:12692. doi: 10.1038/srep12692 (PMC4523848; doi:10.1038/srep12692)
Supplement: Supplementary Information [file srep12692-s1.pdf]

Epitaxial diamond-hexagonal silicon nano-ribbon growth on (001) silicon

Supplementary information : oxidation process and interface structures between diamond-cubic (dc-Si) and diamond-hexagonal (dh-Si) silicon.

Y. Qiu[1,2] , H. Bender*[1], O. Richard[1], M.-S. Kim[1], E. Van Besien[1], I. Vos[1], M. de Potter de ten Broeck[1], D. Mocuta[1], W. Vandervorst[1,2]

[1] Imec, Leuven, Belgium. [2] Instituut Kern-en Stralings Fysika, K.U.Leuven, Leuven, Belgium. Correspondence and requests for materials should be addressed to Y.Q. (email: Yang.Qiu@imec.be) and H.B. (email: hugo.bender@imec.be)

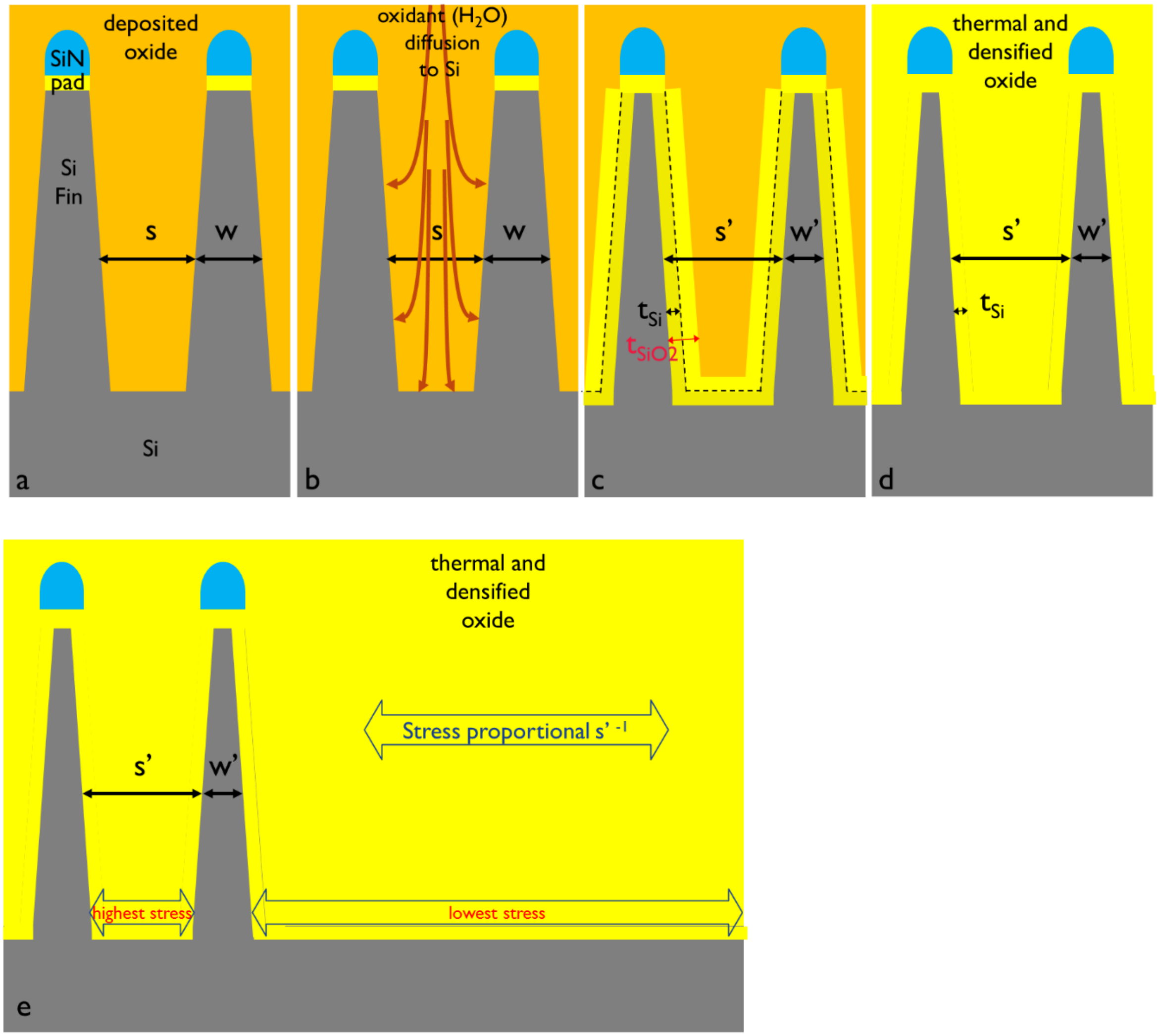

Supplementary Figure S1 | Oxidation process. After filling of the spacing with a chemical vapor deposited oxide (a) a densification oxidation treatment is applied (b). The oxidant (H_2O) diffuses through the deposited oxide and reacts at the Si/oxide interface (b). The thermal oxide grows t_{Si} inside the Si and expands due to the larger oxide volume by a ratio $t_{SiO2}/t_{Si} = 2.2$ (c). Whereas the physical width of the spacings increases from s to s' = s + 2 t_{Si}, the oxide width that has to fit in this spacing increases to s" = s + 2 t_{SiO2}. This volume expansion will be intercepted by the build-up of stress and the possible densification of the deposited oxide. As the oxide is not viscous at the given temperature of the annealing, vertical flow of the oxide will be unimportant and the mechanical stress will be exerted horizontally. During the oxidation step, also the initial thin pad oxide between SiN and Si fin will grow by oxidation of the top of the fin. The oxidation rate of the nitride is much lower so that its width can be used as a marker for the oxidation process (c and Supplementary Fig. S2). Thermal (yellow) and deposited oxide (orange) cannot be distinguished on the TEM images as density/composition are similar and the interface between both oxides will be gradual (d).

As the stress is inversely proportional to s', it is highest at the bottom of the spacings and decreases towards the top (e) and is much lower in the wide open oxide regions (s'=25-35 nm range in standard spacings versus 1-2 µm in the wide regions).

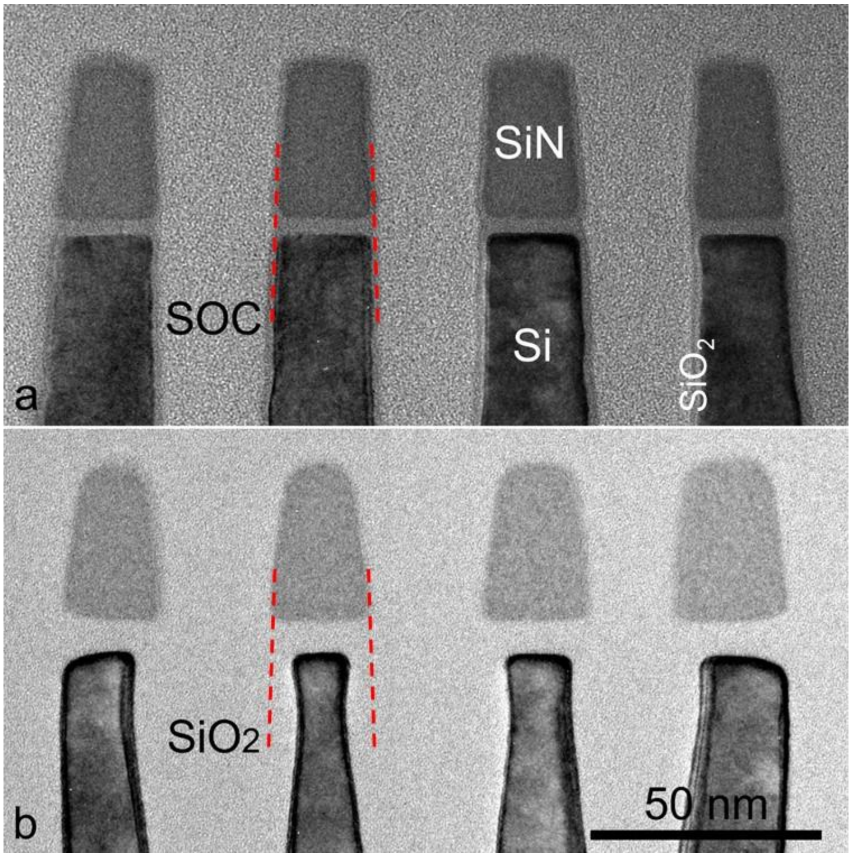

Supplementary Figure S2 | Oxidation process. Transmission electron microscopy images of the 4-fin structure after the etch in the silicon defining the fins (a), corresponding to Figure 1a in the manuscript. The spacings are filled with Spin-on-Carbon (SOC) for the TEM specimen preparation. A thin native oxide (<2 nm) is present on the sidewalls of the Si and SiN. The fin width at the top is similar as the width of the SiN.

After the oxidation treatment (b) the fin width is reduced by the oxidation and the pad oxide thickness is increased (compares to Supplementary Fig. S1d). The oxidation rate of the nitride is much lower so that its width can be used as a marker for the oxidation process. Thermal and deposited oxide cannot be distinguished on the TEM images as density/composition are similar and the interface between both oxides will be gradual.

Interface dc // dh	Distance dc	nm	Distance dh	nm	Ratio dh/dc	Interface structure
(001) // (0-110)	5/2 [110]	1.9192	3 [0001]	1.8951	0.9874	5 and 7 atomic rings in dual plane
(115) // (03-32)	1/2 [552]	1.9944	[01-13]	2.0083	1.0069	5 and 7 atomic rings in single plane
Both interfaces, distance along fin	1/2 [1-10]	0.3838	1/3 [2-1-10]	0.3837	0.9997	

Supplementary Table S1 | Distances between coincident lattice positions in the dc to dh interface and lattice mismatch. Calculated with dc-Si : a=0.54282 nm – dh-Si : a = 0.3837 nm, c = 0.6317 nm[8]. The corresponding interface models are shown in the Supplementary Fig. S3, S4 and S5 below.

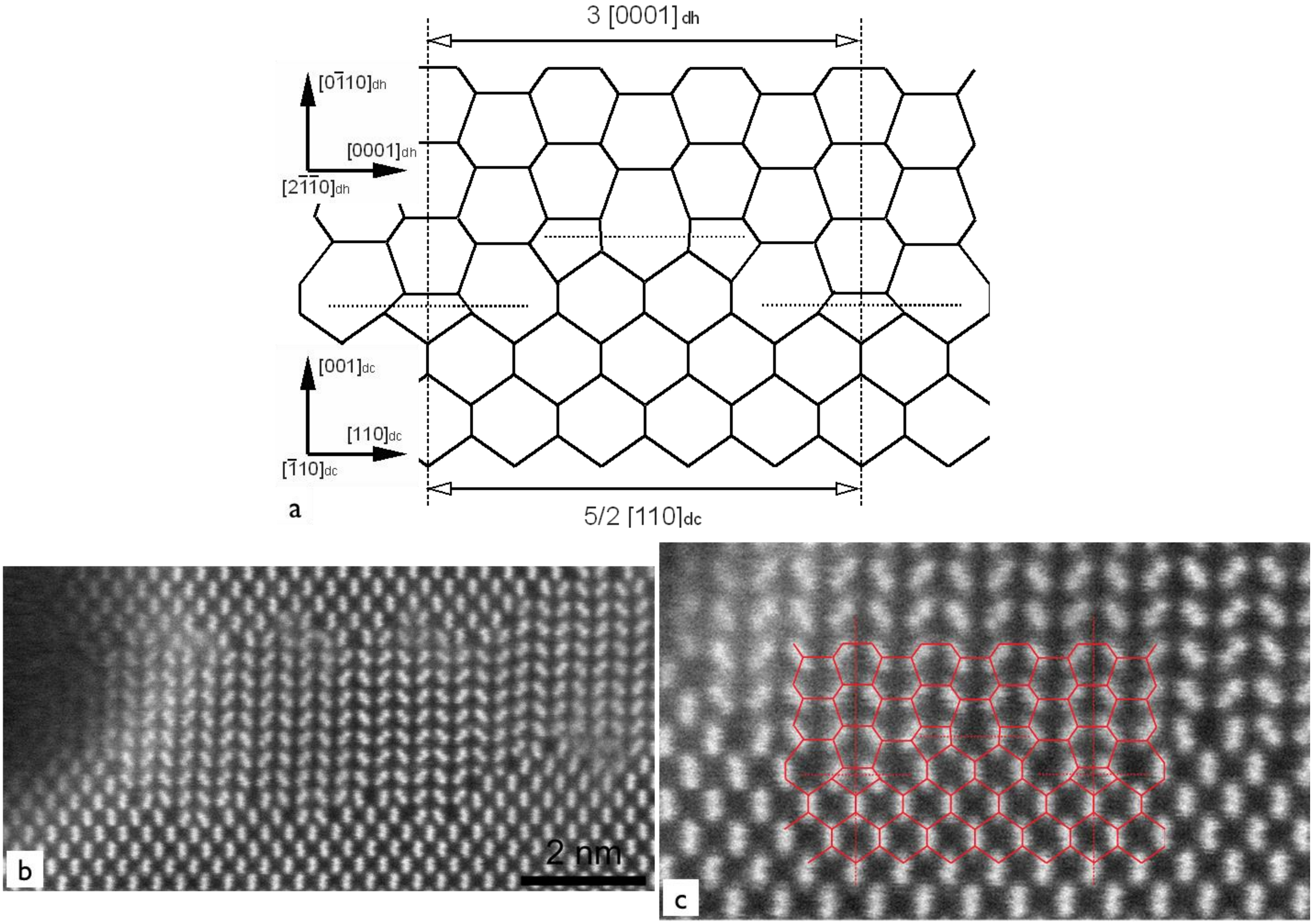

Supplementary Figure S3 | Interface dc-Si/dh-Si with $(001)_{dc}$ // $(0\text{-}110)_{dh}$. The interface can be built with 5 and 7 atomic rings as shown in the model (a). The interface has a periodic stepped structure with two dc rings sticking out as illustrated by the dotted line. I.e. the interface is spread over 2 planes and consists of dc and dh steps. In the upper interface plane a sequence of 2 nearly vertical dumbbells and 4 nearly undistorted tilted dumbbells of the dh lattice are present, while in the lower plane the sequence consists of 2 tilted dumbbells (nearly dh-Si) and 3 vertical dumbbells as in dc-Si. The periodicity in the interface plane corresponds to $5/2[110]_{dc}$ ~ $3[0001]_{dh}$ with 1.26% misfit over a distance of nearly 1.9 nm. The $(0001)_{dh}$ plane is perfectly aligned with the $(110)_{dc}$ plane.

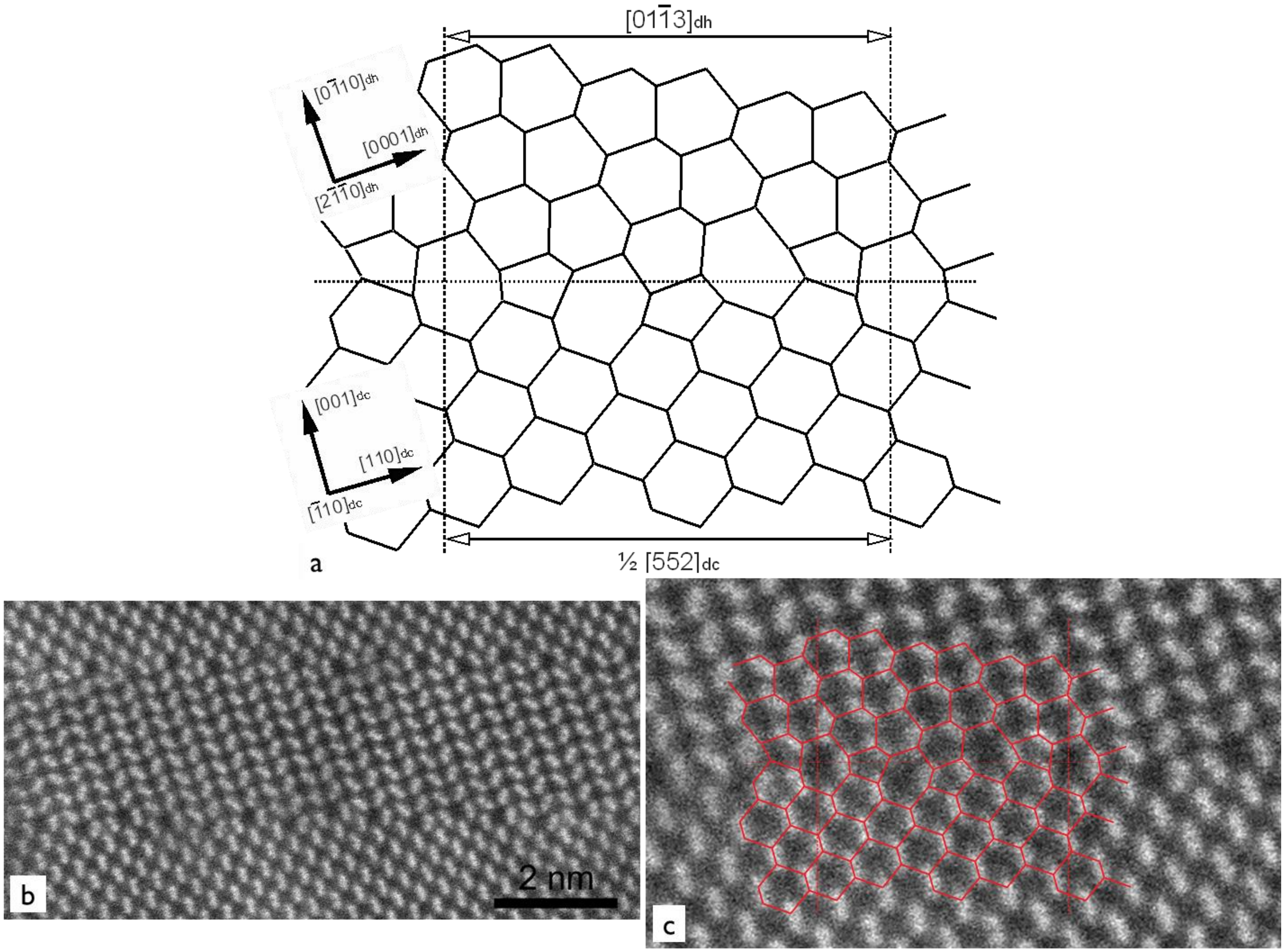

Supplementary Figure S4 | Interface dc-Si/dh-Si with (115)$_{dc}$ // (03-32)$_{dh}$. The interface can be built with 5 and 7 atomic rings as shown in the model (a). The periodicity in the interface plane corresponds to ½[552]$_{dc}$ ~ [01-13]$_{dh}$ with 0.69% misfit over a distance of nearly 2 nm. The model is equivalent with the ones proposed by Tan and Föll[12] and Dahmen et al[13]. The atomic column positions in the HR-STEM image can directly be correlated with the model. The (0001)$_{dh}$ plane is 3.5° rotated relative the (110)$_{dc}$ plane.

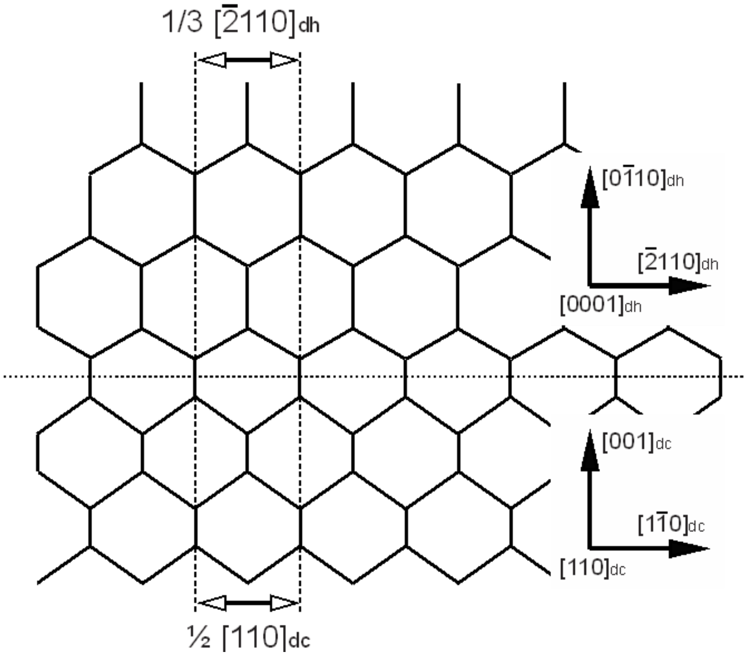

Supplementary Figure S5 | Interface dc-Si/dh-Si with $(001)_{dc}$ // $(0\text{-}110)_{dh}$. Observation along $[110]_{dc}$ as in the TEM samples prepared parallel with the fins. The effect of the dc and dh steps in the dual interface plane (Supplementary Fig. S3) is not taken into account in this schematic.
